# Pregnancy health literacy among teenagers in Kaysone district, Savannakhet Province, Lao PDR

**DOI:** 10.1080/16549716.2020.1791412

**Published:** 2020-08-03

**Authors:** Phonevilai Santisouk, Visanou Hansana, Nguyen Thanh Huong

**Affiliations:** a Faculty of Public Health, University of Health Sciences, Vientiane, Lao PDR; b Institute of Research and Education Development, University of Health Sciences, Vientiane, Lao PDR; c Department of Health Education, Faculty of Social Science, Behavior and Health Education, Hanoi University of Public Health, Hanoi, Vietnam

**Keywords:** LEARN: Sexual Reproductive Health, ANC and Nutrition, Sexuality, teenage pregnancy, health literacy, adolescence, sex education

## Abstract

**Background:**

Pregnancy health literacy (PHL) among teenagers is considered a major protective factor for teenage pregnancy. In Lao PDR, 18% of girls aged 15–19 have begun childbearing and 15% of maternal deaths occur to teenage girls, particularly in rural areas.

**Objective:**

The aim of this study was to describe PHL and its related factors among teenagers in Kaysone district.

**Method:**

This was a cross-sectional study conducted at Oudomvilay and Kheuakhaokat in Kaysone district in January 2019. The Teenage Pregnancy Health Literacy (TPHL) score was collected via face to face interviews covering 33 items with 262 adolescents. Calculation of the TPHL index score was based on the European Health Literacy Survey (HLS-EU) index formula. The TPHL index was also based on the HLS-EU standard level and descriptive statistics were used to explain the score and levels. Descriptive analyses were performed to analyse the individual, family, peer and school variables and to investigate level of TPHL and linear regression was used to identify factors related to TPHL.

**Results:**

The overall score for TPHL was a mean of 27/50. Most (60%) of the adolescents had problematic TPHL levels and only 0.4% had excellent TPHL levels. TPHL was positively and significantly associated with living in urban areas (*β *= *2.42; p* = 0.002), higher education *(β *= 2.93; *p* = 0.004), schooling (*β *= 0.96; *p* = 0.018), being single (*β *= 2.9; *p* = 0.029) and attending classes where sex education content was included (*β *= 4.72; *p < *0.001).

**Conclusion:**

Low TPHL scores show the importance of improving sex education for adolescents as a means of increasing TPHL for better health outcomes in Lao PDR.

## Background

Worldwide, maternal deaths associated with pregnancy and childbirth are significant components of mortality for girls aged 15–19 [1]. Eleven percent of all pregnancies are among adolescents aged 15–19 while one in four women has had a live birth before age 18 [2]. Notably, approximately 95% of these pregnancies occur in low- and middle-income countries [3], where many adolescent girls have difficulties accessing and understanding sexual and reproductive health (SRH) information and are less likely to use contraceptives than adults [4].

Adolescents who had a higher frequency of accessing health information were found to have a higher level of health literacy [5,6]. Inadequate access to health literacy (HL) has been linked to frequent poor health outcomes, such as poor overall health status and higher mortality [7]. HL at an early age can help develop one’s ability to understand health information and improve interactions with the health care system [8]. Maternal health literacy (MHL) is defined as a woman’s knowledge, skills, and ability to gain access to, understand, and use information to promote and maintain her health and that of her children [9]. Pregnancy health literacy (PHL) is defined as the cognitive and social skills which determine the motivation and ability of women to gain and apply the useful knowledge. Education in PHL among teenagers is a major protective factor for teenage pregnancy (TP) [3]. Sexual reproductive health literacy (SRHL) goes beyond knowledge and behaviour and reflects the motivations and competences to access, understand, appraise and apply SRH information to informed decision making. Based on the concept of SRHL [10]. Teenage pregnancy health literacy (TPHL) focuses on the ability of an individual to access, understand, and appraise and apply the information into informed decision making for teenage pregnancy prevention.

Lao PDR is a lower-middle income country in South East Asia [11,12], with 60% of the population estimated to be under aged 25 [13]. The health system is largely public and is characterized by low Government spending, high out-of-pocket expenditure, low coverage and poorly resourced facilities [11,12]. Societies that are strict about preventing sexual activity and pregnancy outside marriage restrict behaviour through social attitudes [10,14]. The legal age of marriage for adolescents is 18 years. However, it is common for the law to allow underage marriage in special and necessary cases, such as cases of under-age pregnancy [15,16]. The culture of girls marrying early is advantageous for the recipient family as they gain a labourer [15]. Teenage pregnancy (TP) is still an important public health problem. According to the Lao Statistics Bureau (LSB) and Ministry of Health (MOH), 15% of maternal deaths are teenagers and 18% of girls aged 15–19 had begun child bearing, particularly in rural areas [17]. Maternal mortality rate is high, at 197 deaths per 100,000 live births [13]. Seventy percent of the young population reside in rural areas with the largest number in the major provinces of Savannakhet, Vientiane and Champasak [18].

The low general HL [19] and high rate of TP in Lao PDR indicates that there is a lack of effective SRH education among adolescents. This can be partly related with low rates of school exposure to sex education at school [3,17,20]. Adolescents who study in rural areas had a lower sexual and reproductive health literacy (SRHL) score (16/50) and those who rarely or never attended SRH subject classes regularly had lower SRHL (17/50) [10]. A previous study emphasizes the importance of quality, curriculum-based sexual education (CSE) programmes, but they are not available to every grade in rural and urban areas. Attempts are being made to use this programme with every grade, incorporated into the curriculum. However, adolescents who are not in school can find information outside school from Youth Friendly Services (YFS) [10]. The CSE is being integrated into school curricula, currently integrated in biology, which is taught in year six upper secondary school. To date, there is no CSE as a separate subject in the school curriculum in Laos [10,21,22].

Therefore, SRH education is a critical factor as a low level of SRH education has been identified as a contributing factor to adolescent pregnancy [23].The higher the level of education one attains, the higher the level of HL. Further, access to reproductive health information has been found to have a positive impact on HL in adolescents [24].

Until now, research has focused on the situation of HL and its relation to adverse health behaviour and outcomes [25]. This, however, has also created a dearth of information regarding the current situation of TPHL and related factors among teenagers aged 15–19. This study was conducted to describe current TPHL and related factors among teenagers (15–19 years old) in Kaysone district, Savannakhet Province, Lao PDR. Findings from this research can help health providers and other stakeholders to improve reproductive health services for this target population.

## Methods

### Study setting and design

This quantitative cross-sectional study was designed to use face-to-face interviews to assess the self-capacity of adolescents in Oudomvilay and Kheuakhaokat village, Kaysone district in January 2019. With a population of 130,000, Kaysone district is the second-largest in Laos PDR after Vientiane, with 31 villages in urban areas and 36 villages in rural areas, some with road access and some without. Despite being near the capital city, some villages lack basic infrastructure such as piped water, regular markets or passable roads. Only this district in the province, and one in Vientiane, has a Youth Friendly Service (YFS), which has been run since 2012 by MOH with support from United Nations Population Fund (UNFPA). With a YFS, one would expect that there would be consultation services, information provided in schools and in the community about SRH, including the topics of pregnancy and contraception, and also providing health services for adolescents. However, there had been no formal surveys or research in this district related to SRH. Hence people in the health service departments in Savannakhet and I wanted to conduct this study in the district to provide an empirical basis for knowledge and measures taken regarding SRH among its young people as well as for an assessment of the of the comprehensiveness and effectiveness of the services of the YFS.

### Study sample and sampling

Adolescents aged 15–19, both male and female, unmarried and married, schooling and non-schooling, were the study population. Younger adolescents aged 13–14 years and young Buddhist monks or novices and adolescent who moved to the district recently were excluded from this study as it was thought to be inappropriate to ask them about sexuality and because they were not representative of the general youth in this study. For the sample size, a calculation was made based on the estimation that teenagers aged 15–19 made up 10% of everyone living in Kaysone district [17]. The sample size was then determined by using a formula on this total target population of 13,000 young people whereby 50% of the adolescents were estimated to have inadequate levels of TPHL, a confidence level of 95% should be applied and the result was a critical sample size of 262 adolescents aged 15–19. A sample of 262 adolescents was drawn through multi-stage sampling from 332 adolescents in the two villages.

**Figure 1.**
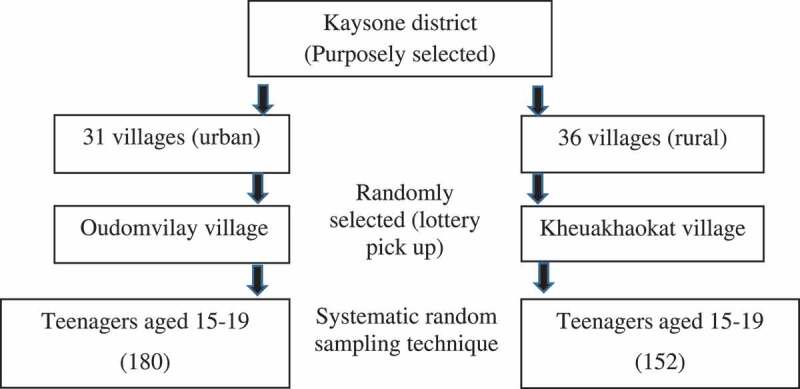
Diagram of sampling method.

Savannakhet Province has 15 districts (67 villages), from which Kaysone district was purposely selected because of the existence of the YFS there. Two villages from this district was randomly selected (‘lottery’ pick up). A list of all adolescents aged 15–19 living in these two villages was obtained, upon which a systematic random sampling technique was used to select the study participants (Figure 1).

**Figure 2.**
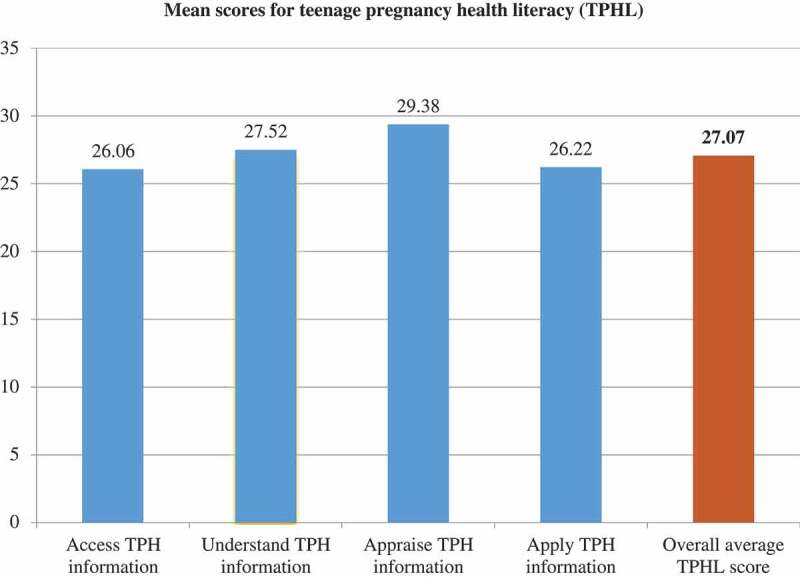
Mean score of teenage pregnancy health literacy.

### Data collection

Data were collected by women (interviewing girls) and men (interviewing boys) who worked in the health care service. Both men and women were engaged as interviewers due to the sensitive information related to adolescents, and an extensive one-day training session was held to standardize the data collection procedures. The training included a review of the study objectives, the legal context and its implications for the confidentiality of research participants, interpersonal issues, ethics, confidentiality, informed consent, interviewing techniques and detailed instructions on the structured TPHL questionnaires.

### Variables

Individual traits plus peer, parental and system factors all combine to influence one’s health literacy. Three groups of independent variables were included in this study: individual variables (age, sex, area, education, status of school, marital status and living status), family, peer variables (education of mother and father, occupation of mother and father, family income and talking about pregnancy or contraception with friends), and school variables (attending classes where sex education content is included and number of courses or activities related to sex education one month before). The one dependent variable was pregnancy health literacy measured by the TPHL scale mentioned above.

### Research instrument

A structured questionnaire consisted of two parts was used: TPHL and individual, family, peer and school information. Based on a previous study by Vonxay [10], which included pregnancy, contraception and abortion, this study focused on pregnancy and contraception among a sample of adolescents in community. In this study, the validity of the questionnaire was first tested in a pilot survey among 40 adolescents in Vientiane. Completing the questions took the participants 30–35 minutes. The design was assessed as complete and sufficient; only small adjustments were made. The response rate for the TPHL questions was good. Internal consistency was satisfactory at 0.85 on Cronbach’s alpha [26]. The data of TPHL among population are normally distributed.

TPHL level was measured using 33 items under four components: access (7 items), understanding (8 items), appraisal (6 items) and application (12 items) for health-related information on a 4-point Likert scale: 1 = very difficult, 2 = fairly difficult, 3 = fairly easy and 4 = very easy.

We calculated the TPHL score using the formula ‘Index score = (mean – 1)*(50/3)’. The measurements were divided into four categorical levels: Inadequate literacy, problematic literacy, sufficient literacy and excellent literacy. The cut-off points of these categories follow those categorical of the TPHL, inadequate: 0 to 25, problematic: 26 to 33, sufficient: 34 to 42 and excellent: 43 to 50 Both the formula and the scales were adopted from the European health literacy survey (HLS-EU-Q47) method [27].

### Statistical analysis

This study used STATA v 14.2 to analyse the data. Descriptive analyses were performed to analyse the individual, family, peer and school variables and to investigate the level of TPHL. Linear regression was used to identify the factors related to TPHL. Comparisons between groups were made with Chi-square test for independence, t-tests (2 variables) and one-way ANOVA (more than 2 variables). Bivariate and multivariate linear regression models were used to identify predictors of TPHL. Only factors with a *p* < 0.05 on these tests were included in the multiple linear regression model. For this model, a backward elimination strategy was used, with p = 0.05 as the cut-off level of significance. Linear regression results were presented as unstandardized regression coefficients.

### Ethical considerations

The ethic clearance was obtained from the National Ethics Committee for Health Research, Ministry of Health, Lao PDR and the International Review Board of the Hanoi University of Public Health. Verbal and written consent was obtained from the father, mother or guardian of each participant prior to interviews with the approval of the National Ethics Committee for Health Research. Due to the sensitive nature of the topics in the questionnaire, participants were informed about confidentiality agreements to ensure their privacy. Participants were guaranteed anonymity during and after the research. When conducting the interviews, the interviewers did not record the names and addresses of participants but only their participant identification numbers.

## Results

A total of 262 teenagers completed the questionnaire, with no teenagers in attendance refusing to participate. Thirty-five percent of respondents had had sex at least once, 19 reported that they or their girlfriend became pregnant (five boys and 14 girls) and two girls were currently pregnant. The overall score for TPHL had a mean of 27/50 (Figure 2). Most of the adolescents scored within the ‘problematic’ TPHL level, ranging between 26 and 33 based on the HLS-EU interpretation. Further inspection revealed that 158 had TPHL scores in the ‘problematic’ range (59.9%) and only one respondent (0.4%) showed ‘excellent’ TPHL. The result showed 57.2% of participants found it difficult to find information about activities that they could join regarding contraceptives, teenage pregnancy. However, approximately 60.0% felt it was easy to judge the quality of information from their family and friends about pregnancies and contraceptives. Almost 67% of teenagers found it difficult to understand what to do in case they/their girlfriend had a dangerous problem related to pregnancy such as unsafe abortion, young mother death, violence and sexually Transmitted Infections (STIs) including HIV/AIDS [28]. Almost 66.4% of the respondents found it difficult to decide what to do when they or their girlfriend had a problem related to pregnancy.

Table 1 presents the relationship between PHL among teenagers aged 15–19 and individual factors. This study revealed that there was a statistically significant relationship between adolescents’ education and TPHL level *(p < *0.001). There was a significant association between the between the marital status of teenagers and TPHL level (*p < 0.001*). In addition, significant associations were found between father’s and mother’s education and TPHL level, respectively at (*p = *0.035 and *p = *0.038) and father’s and mother’s occupation and TPHL level, respectively at (*p < 0.001* and *p < 0.001*). There was a significant association between attendance at classes where sex education content was included and TPHL level (*p < *0.001): participants who had attended these classes had a higher TPHL level (28/50) than those who had not attended (20/50). In this study, no significant associations were found between TPHL scores and age, gender, living status of adolescent, family income and marital status of parent and number of participants who had taken a course or activity related to sex education in the previous month.

**Table 1.** Individual, family, peer and school factors related to TPHL score.

	Total teenagers n=262	TPHL score	
Characteristics of teenagers variables	Freq. (%)	Mean	p-value
**Individual**			
**Age (years)** Mean = 17.66	-	-	-
Mean = 17.66 ± 1.3, min = 15, max = 19,			(0.156)
**Sex**	0.078		
Male	99(38)	27.8	
Female	163(62)	26.6	
**Living area**			<0.001*
** **Rural	114(43.5)	24.7	
Urban	148(56.5)	28.9	
**Schooling status**	<0.001*		
Out of school	51(19.5)	24.3	
Lower/Upper secondary school/University	27(10.3)	27.7	
**Highest level of education completed:**			<0.001*
Primary school	46(17.5)	21.8	
Lower	122(46.6)	27.5	
Upper secondary school	94(35.9)	29.3	
**Marital status**			<0.001*
Single	237(90.5)	27.6	
In-union	4(1.5)	22.4	
Married	19(7.2)	21.9	
Divorced/separated	2(0.76)	23.7	
**Living status of adolescent**	0.56		
Living with parent	219(83.6)	27	
Living with sibling/spouse/alone	43(16.4)	27.5	
**Family**			
**Father’s occupation**			<0.001*
Low income (farmer, laborer)	177(67.6)	26.2	
High income (Gov./private staff Merchant)	85(32.4)	28.7	
**Father’s educational level**			0.035*
Illiterate	23(8.8)	25.1	
Educated	239(91.2)	27.2	
**Mother’s occupation**			<0.001*
Low income (farmer, laborer)	179(68.3)	26.2	
High income (Gov./private staff Merchant	83(31.7)	28.8	
**Mother’s educational level**			
Illiterate	38(14.5)	25.6	0.038*
Educated	224(85.5)	27.3	
**Family income ^(a)^**			
Mean = 265 ± 187, min = $11.7, max = $1,176,	0.1		
**Peer**			
**Talk about sexual health (pregnancy/ contraception) with friend**	0.731		
** **No	176(67.2)	27.2	
Yes	86(32.8)	26.7	
**School**			
**Attended classes where sex education content was included**			<0.001*
No	33(12.6)	20.4	
Yes	229(87.4)	28.0	
**Number of times participant had taken a course or activity related to sex education in the month before**	0.104		
Nil/1 time	234(89.3)	26.8	
More than 1 time	28(10.7)	28.6	

(a) Mean age of teenagers was 265 ± 187, min = 11.7 max = 1176. Based on spearman’s correlation, family income wasn’t found to be correlated with TPHL score.

(*) The p-value of less than 0.05 was used to determine the statistical significance of the tests with a regression coefficient to predict the strength and direction of the association.

The significant factors with a *p* < 0.05 on these tests were included in the multiple linear regression model [29]. Five independent variables (indicated by **) had significantly correlated with TPHL, with *p* < 0.05. Table 2 showed that TPHL was associated with living in urban areas (*β *= *2.42; p* = 0.002), higher education *(β *= 2.93; *p* < 0.004), schooling (*β *= 0.96; *p* = 0.018), being single (*β *= 2.9; *p* = 0.029) and attendance at classes where sex education content was included (*β *= 4.72; *p < *0.001). However, father’s and mother’s occupation, father’s and mother’s education were not significant direct predictors of TPHL scores in this study. Table 2 shows more details.

**Table 2.** Factors associated with level of TPHL in Kaysone district.

			95%CI	
Variables	Regression coefficient (β)	p-value	Lower	Upper
**Individual**				
**Living Area**				
Rural	Ref.			
Urban	2.42	0.003**	0.879	3.962
**Highest level of education completed:**				
None/Primary school	Ref.			
Lower/Upper secondary school	2.93	<0.004**	0.45	2.293
**School status**				
Out of school	Ref.			
Schooling	0.96	0.018*	0.426	4.495
**Marital status**				
Married/divorced/separated/in-union	Ref.			
Single	2.9	0.029*	0.248	4.608
**Family**				
**Father’s education **				
** ** Illiterate	Ref.			
Educated	0.27	0.785	-1.214	1.605
**Fathers’ occupation**				
** ** Low income (farmer, laborer)	Ref.			
High income (Gov./private staff Merchant	0.32	0.747	-1.816	2.53
**Mother’s education **				
Illiterate	Ref.			
Educated	0.19	0.849	-1.599	1.942
**Mothers’ occupation**				
** ** Low income (farmer, laborer)	Ref.			
High income (Gov./private staff Merchant	1.73	0.08	-1.167	2.586
**School**				
**Attend the class where sex education content included**				
No	Ref.			
Yes	4.72	<0.001**	3.367	8.184

*significant association (*p* < 0.05).

**significant association (*p* < 0.01).

## Discussion

This study described TPHL among adolescents 15–19 years old in Kaysone district, Savannakhet Province, Lao PDR and associated factors. The results in our study showed that most participating adolescents had a ‘problematic’ TPHL level. As this was the first time TPHL was measured and comparative literature was lacking, the most closely related parameter SRH, used by a previous study [10], was applied in this study. Overall, 89.7% (n = 235) of teenagers were found to have a ‘problematic’ or less than sufficient TPHL on the index. However, participants were qualitatively interviewed and from the interviews were able to demonstrate ability in accessing, understanding, appraising and applying health information. This study also found that the four components were less than sufficient TPHL. These scores indicated that the participants did not possess the competence to maintain and improve their quality of life [25].

In contrast, the previous study by Vongxay et al [10] discovered inadequate SRHL among adolescents aged 15–19 and looked at schooling adolescents in three provinces with only Vientiane capital running a YFS. It could be explained that the slightly higher TPHL among teenagers was due to the YFS implemented in schools and the community, providing information about SRH (including pregnancy and contraception) and health services for adolescents. Therefore, adolescents there had access to resources at the YFS in addition to access to other information sources such as libraries, print media, television, radio and the Internet. Such access could have had an impact on the literacy patterns and the overall health of the society. Even if the 15 to 19-year age range in this study meant that the younger respondents would not have received sex education in school and might not have started sexual activity, educational levels in the sample population were probably the most relevant factor in TPHL.

In terms of predictive factors for TPHL levels, seven significant ones were identified: living area, education, schooling status, marital status, father’s education, mother’s education and attendance of classes with sex education content. Apart from father’s occupation and mother’s occupation which proved to be not significant factors for TPHL levels in this study, individual, family, peer and school factors contributed to the predictive model. This finding was consistent with those in the literature [10,19,30–32]. In this study, no significant associations were found between TPHL level and age. Those previous studies as well as this study did not found associations between age and HL because the sample of teenagers was smaller in age range [10,31,33].

Another finding of this study is that more than half of the adolescents with a high TPHL level lived in an urban area (56.5%). This is consistent with the previous study [10], which indicated that adolescents who lived in urban areas showed a significant association with SRHL level (*β = *3.21: *p < *0.001). Their peers living in rural areas, on the other hand, were more likely to have low HL [31]. It may be inferred that urban conditions facilitate access to information, which in turn enables better care, treatment, health protection and health promotion than in rural areas. Living area, whether urban or rural, is thus considered a factor strongly associated with HL. Being healthy suggests having the opportunity to recognize health problems, which comes with access to health information, including SRH and thereby PHL.

This study revealed that there was a statistically significant relationship between adolescents’ education and TPHL, which was corroborated by previous studies’ finding that adolescents with a lower educational level were associated with lower estimated HL [31,33]. In general, level of education is a factor associated with ‘basic literacy skills’, and in a study by Wallace, those who had a higher level of education were found to score higher on health literacy [23]. Higher education also comes with more knowledge on sexuality and reproductive health. In Savannakhet, the Ministry of Education and Sports have been able to provide reproductive health education at the end of the course. All considered, it means that higher education itself is a major protective factor for teenage pregnancy: more years of schooling is associated with less teenage pregnancies [3].

Adolescents who are in school have a greater chance of higher TPHL scores. In a study by Martin et al., adolescents who were still in lower primary school or in General Education Development were associated with lower estimated health literacy (*p < *0.01) [31]. Given that literacy, or the ability to read and write, is an integral part of health literacy, schools therefore play a central role in the development of TPHL skills. With school-based comprehensive sexuality education programmes, teachers have an opportunity to encourage adolescents to delay sexual activity and encourage them to behave responsibly when they eventually engage in consensual sexual activity, particularly by using condoms and other modern methods of contraception [32].

In this study, the adolescents who were single were more likely to have higher TPHL score (28/50) than others (22/50) while previous studies found that individuals who were not married also had lower HL, on average, although the association was much weaker [31].The difference in this study could be explained that single teenagers had greater opportunity to study in school and thus to receive sexual health information and teenage pregnancy health information. Moreover, this research showed that most of the adolescents who were single had attended classes in school with sex education content included.

Attendance at classes where sex education content is included is one of the key factors in TPHL because such participation exposes the adolescents to specific SRH matters related to pregnancy and contraception. The finding of this study showed that almost all of the participants had attended classes with some sex education content (89.3%). The relationship between such class attendance and TPHL score was significant. Similar to the findings of Vongxay [10], this research found that there was an association between TPHL score and attending these classes. Such education on sexuality is more likely to have a positive impact when it is comprehensive and implemented by trained educators. In these classes, educators had the opportunity to encourage adolescents to delay sexual activity and to behave responsibly when they eventually engage in consensual sexual activity [32]. Such input from these educators can have an impact on literacy patterns and society’s health at large.

## Study limitations

This study also has some limitations. First, participant recruitment and data collection were confined to one geographical area. This means that the sample may not be representative of all the adolescents in Lao PDR and does not address different contexts. This study is of a cross-sectional design also, prevents us from assessing the temporal order of TPHL. In addition, a questionnaire on TPHL would elicit rather sensitive information of the issue among adolescents. Culturally, adolescents had premarital sex is illegal by school regulations, and could be in being excluded, respondents might have given socially desirable answers to the question linked to sexual experience. This study was to ensure privacy during answering of the questions by giving enough physical space in between each participant and requesting silence in this age group. Additional limitations were timeframe of the study.

## Conclusion

From this study, most teenagers had either a problematic (60%) or an inadequate TPHL level (30%). Lower levels of TPHL were found among teenagers with less education, living in rural areas, out of school, no longer single and who did not attend classes where sex education was included. These teenagers also tended to have lower scores if their fathers and mothers had lower education. The study also identified five predictors that were significant (*p < *0.05), namely, living area, education, marital status and status of school as individual factors, father’s and mother’s education as parental factors and having attended classes where sexual education content was included as school factors.

This research demonstrates the need for more teacher training and the development of sustainable sex education programmes (with a focus on pregnancy prevention) in schools. In the health sector, there is an ongoing need to enhance the availability of YFS for pregnancy prevention. In addition, there is a need to strengthen the capacity of service providers to respond to specific needs of adolescents more effectively with greater sensitivity. Parents should be educated on the importance of offering helpful advice for pregnancy prevention to their adolescents and giving correct information on pregnancy prevention issues according to their ages. Future qualitative research should be conducted to get in-depth insights into parents’ and adolescents’ specific problems and TPHL.

## Supplementary Material

Supplemental Material

## Paper context

Teenagers aged 15–19 are considered key and vulnerable populations with regards to teenage pregnancy. This study does not only indicate that teenagers have a ‘problematic’ or less than sufficient TPHL on the index, which means adolescents lack of capacity to access, understand, appraise and apply SRH information, but the low TPHL scores also indicates that adolescents didn’t possess the competence to maintain and improve their quality of life.
