# Supplementary material for: Pregnancy health literacy among teenagers in Kaysone district, Savannakhet Province, Lao PDR
Source: Glob Health Action. 2020 Aug 3;13(Suppl 2):1791412. doi: 10.1080/16549716.2020.1791412 (PMC7480437; doi:10.1080/16549716.2020.1791412)
Supplement: Supplemental Material [file ZGHA_A_1791412_SM8930.docx]

**Questionnaire**

**Teenage Pregnancy Health Literacy for Lao teenagers in Kaysone district, Savannakhet Province, Lao PDR**

(Questionnaire for interview surveys with young people)

**ID: ………………………….**

Please answer these questions by filling in the answers or putting a check **(√)** against the answers accordingly, based on truthful information about yourself, If you have filled in a wrong answer, use a big cross (X) for the wrongly indicated answer and use circle the correct answer.

**Part 1: Independent variables: individual, family or peer and school information**

| **Individual** | | |
| --- | --- | --- |
| Q1.1 | What is your date of birth?  What is your age? | Date of birth…/…../……(dd/mm/ yyyy)  Age………..years |
| Q1.2 | What is your sex? | □ 0. Male □ 1. Female |
| Q1.3 | What is your place of birth? | District…………… Province……………..  Country………………………. |
| Q1.4 | Where do you currently live? | Village…………………………. |
| Q1.5 | Which of the following best describes the area you live in? | □ 0. Rural □ 1. Urban |
| Q1.6 | What is your ethnicity? | □ 1. Lao tai  □ 2. Hmong-mien  □ 3.Mon-Keummu  □ 4. Chinese tibetan  □ 99. Other specify………….. |
| Q1.7 | What is your primary language? | □ 1. Lao  □ 2. Minority ethnic language  □ 99. Other specify………………….. |
| Q1.8 | What is your religious belief? | □ 1. Buddhism  □ 2. Spiritual belief  □ 3. Islam  □ 4.Christian  □ 99. Other specify………… |
| Q1.9 | What is the highest level of education you have completed?  ***If “None/…” skip to Q1.11*** | □ 0. None/never went to school  □ 1. Primary  □ 2. Lower secondary school  □ 3. Upper secondary school  □ 4. Vocational school  □ 5. Higher education (university) |
| Q1.10 | What is your schooling status? | □ 0. Out of school  □ 1. Primary  □ 2. Lower secondary school  □ 3. Upper secondary school  □ 4. Vocational school  □ 5. Higher education (in university)  □ 99. Other (please specify)………… |
|  | Q1.10a Which level are you in? | ……………………………… |
| Q1.11 | Do you have a job? | □ 0. No  □ 1. Yes |
| Q1.12 | What is your job? | □ 1. Student  □ 2. Gov./private staff  □ 3. Laborer  □ 4. Farmer  □ 5. Merchant  □ 99. Other (please specify)………… |
| Q1.13 | What is your relationship status? | □ 1. Single  □ 2. In a relationship/in union  □ 3. Married  □ 4. Divorced/separated  □ 99. Other (please specify)………… |
| Q1.14 | Have you had sexual intercourse? | □ 0. No **if No skip to q1.17**  □ 1. Yes |
| Q1.15 | Do you have children? | □ 0. No  □ 1. 1 child  □ 2. 2 children  □ 3. 3 or more |
| Q1.16 | For girls, are you currently pregnant? | □ 0. No  □ 1. Yes |
| Q1.17 | Who are you living with? | □ 1. living with parent  □ 2. living with relatives  □ 3. living with spouse/partner  □ 4. living alone |
| **Family** | | |
| Q1.18 | Is there anyone in your family who works in a medical-related career? | □ 0. Nobody  □ 1. Yes, father  □ 2. Yes, mother  □ 3. Yes, spouse  □ 4. Yes, sibling |
| Q1.19 | What is the marital status of your parents? | □ 1. Married  □ 2. Divorced  □ 3. Separated  □ 4. Widowed /Widowered |
| Q1.20 | What is your father’s occupation? | □ 1. Gov./private staff  □ 2. Laborer  □ 3. Farmer  □ 4. Merchant  □ 99. Other (please specify)………… |
| Q1.21 | What is the highest level of education your father has completed? | □ 0. Never went to school  □ 1. Primary  □ 2. Lower secondary  □ 3. Upper secondary  □ 4. Vocational school  □ 5. Higher education (university)  □ 99. Other (please specify)………… |
| Q1.22 | What is your mother’s occupation? | □ 1. Gov./private staff  □ 2. Laborer  □ 3. Farmer  □ 4. Merchant  □ 99. Other (please specify)………… |
| Q1.23 | What is the highest level of education your mother has completed? | □ 0. Never went to school  □ 1. Primary  □ 2. Lower secondary  □ 3. Upper secondary  □ 4. Vocational school  □ 5. Higher education (university)  □ 99. Other (please specify)………… |
| Q1.24 | What is your current combined household income per month? (of all persons living in your household) | □ ……………………..$  (Estimate in Lao kip the total income from all persons living in your household) |
| **Peers** | | |
| Q1.25 | Do you talk with friends about sexual health (pregnancy/ contraception…)? | □ 0. No  □ 1. Yes |
| **School** | | |
| Q1.26 | Have you attended classes where sex education content was included? | □ 0. No ***Skip to q1.29***  □ 1. Yes |
| Q1.27 | What the sex topic(s) did education content cover in the class (es) that you had in school?  **(Tick more than one )** | □ 1.Contraception  □ 2. Pregnancy, maternal & child health care  □ 3. Changes during puberty  □ 4. STIs (HIV/AIDs)  □ 5.Families, gender and human rights  □ 99 Others (please specify)…………. |
| Q1.28 | Which subject(s) in school have included sex education content? | □ 1. Biology  □ 2. Geography  □ 3. Population sciences  □ 4. World around us  □ 99. Others (please specify)………… |
| Q1.29 | Have you taken a course or participated in an activity related to sex education within the last month? | □ 0. No/1 time  □ 1. More than 1 time |

**Part 2: Dependent variable: Teenage Pregnancy Health Literacy**

This part of the questionnaire is used to measure the teenage pregnancy health literacy based on the self-perception of the respondents. Some questions in this section may have to be assumed (according to the interviewer’s training) for those who have never had sexual experience, to comfortably answer the question.

| On a scale from ‘very easy’ to ‘very difficult’, how easy would you say it is to: … | | **Circle the answer number** | | | | | | |
| --- | --- | --- | --- | --- | --- | --- | --- | --- |
|  |  | Very  Difficult | Difficult | | Easy Very | | Very  Easy | |
| **Accessing** | | | | | | | | |
| 2.1 | …find information about which contraceptive you can use? | 1 | 2 | | 3 | | 4 | |
| 2.2 | …find information about possible side-effects of contraceptives? | 1 | 2 | | 3 | | 4 | |
| 2.3 | …find information about early symptoms of pregnancy and pregnancy testing? | 1 | 2 | | 3 | | 4 | |
| 2.4 | …find information about how you can live healthily during a pregnancy? | 1 | 2 | | 3 | | 4 | |
| 2.5 | …find information on where to get (professional) help when you are/your girlfriend is pregnant? | 1 | 2 | | 3 | | 4 | |
| 2.6 | …find information about problems that can occur during a teenage pregnancy? | 1 | 2 | | 3 | | 4 | |
| 2.7 | …find information about activities (in your community or school) that you can join about contraceptives, teenage pregnancies? | 1 | 2 | | 3 | | 4 | |
| **Understanding** | | | | | | | | |
| 2.8 | …understand your doctor’s/pharmacist’s instructions on how to use contraceptives/medicine? | 1 | 2 | | 3 | | 4 | |
| 2.9 | …understand information that comes with your leaflet/product packages (e.g. condom or medicine packaging). | 1 | 2 | | 3 | | 4 | |
| 2.10 | …understand information in the media (e.g. Facebook, Google, television, brochures, and posters) about pregnancy, contraceptives? | 1 | 2 | | 3 | | 4 | |
| 2.11 | …understand how sexual intercourse without contraceptive methods (e.g. condoms, etc.) can lead to pregnancy? | 1 | 2 | | 3 | | 4 | |
| 2.12 | …understand how you can test if you are/your girlfriend is pregnant and what symptoms occur in the first stage of pregnancy? | 1 | 2 | | 3 | | 4 | |
| 2.13 | …understand why pregnant teenage girls need to live healthily and see a professional doctor regularly during their pregnancy? | 1 | 2 | | 3 | | 4 | |
| 2.14 | …understand what to do in case you have/your girlfriend has a dangerous problem related to pregnancy? | 1 | 2 | | 3 | | 4 | |
| 2.15 | …understand the problems that can occur if you are/your girlfriend is pregnant? | 1 | 2 | | 3 | | 4 | |
| **Appraising / Thinking** | | | | | | | | |
| 2.16 | …judge what the advantages and disadvantages are for you of using contraceptives? | 1 | | 2 | | 3 | | 4 |
| 2.17 | …judge if it is necessary for you to go to a doctor if you have questions about pregnancies, contraceptives? | 1 | | 2 | | 3 | | 4 |
| 2.18 | …judge the quality of information in the media about pregnancies, contraceptives? | 1 | | 2 | | 3 | | 4 |
| 2.19 | …judge the quality of information from your family and friends about pregnancies, contraceptives? | 1 | | 2 | | 3 | | 4 |
| 2.20 | …judge the quality of information from your doctor or pharmacist about pregnancies, contraceptives? | 1 | | 2 | | 3 | | 4 |
| 2.21 | …judge the quality of information from your teacher or out-school/work activities? | 1 | | 2 | | 3 | | 4 |
| **Applying** | | | | | | | | |
| 2.22 | …use contraceptives before having sex? | 1 | | 2 | | 3 | | 4 |
| 2.23 | …use the doctor’s or pharmacist’s instructions about pregnancies, contraceptives? | 1 | | 2 | | 3 | | 4 |
| 2.24 | …follow the instructions that come with your leaflet/product packages (e.g. condom or medicine packaging)? | 1 | | 2 | | 3 | | 4 |
| 2.25 | …decide what to do when you discover/your girlfriend discovers a pregnancy? (Keeping the baby, adoption)? | 1 | | 2 | | 3 | | 4 |
| 2.26 | …decide what to do when you have/ your girlfriend has a problem related to pregnancy? | 1 | | 2 | | 3 | | 4 |
| 2.27 | …decide what to do when you have/ your girlfriend has a problem related to contraceptives? | 1 | | 2 | | 3 | | 4 |
| 2.28 | … talk/negotiate with your sexual partner about using a contraceptive method before having sex? | 1 | | 2 | | 3 | | 4 |
| 2.29 | …talk with your friends about teenage pregnancies, contraceptive methods? | 1 | | 2 | | 3 | | 4 |
| 2.30 | …talk with your family about teenage pregnancies, contraceptive methods? | 1 | | 2 | | 3 | | 4 |
| 2.31 | …consult your health provider (e.g. doctor or nurse) about teenage pregnancies, contraceptives? | 1 | | 2 | | 3 | | 4 |
| 2.32 | …consult your teacher about teenage pregnancies, contraceptives? | 1 | | 2 | | 3 | | 4 |
| 2.33 | … join/take part in sexual education activities, family planning, and teenager supportive activities | 1 | | 2 | | 3 | | 4 |
